# Lack of Genetic Influence on the Innate Inflammatory Response to *Helicobacter* Infection of the Gastric Mucosa

**DOI:** 10.3389/fimmu.2012.00181

**Published:** 2012-07-04

**Authors:** John G. Nedrud, Steven J. Czinn, Hua Ding, Brandon M. Zagorski, Raymond W. Redline, William Twaddell, Thomas G. Blanchard

**Affiliations:** ^1^Department of Pathology, Case Western Reserve UniversityCleveland, OH, USA; ^2^Department of Pediatrics, University of MarylandBaltimore, MD, USA; ^3^Institute for Clinical Evaluative SciencesToronto, ON, Canada; ^4^Department of Pathology, University of MarylandBaltimore, MD, USA

**Keywords:** *Helicobacter pylori*, inflammation, genetics, SCID, gastritis

## Abstract

*Helicobacter pylori* (*H. pylori*) is a bacterial pathogen that resides at the gastric mucosa and has a world-wide prevalence of over 50%. Infection usually lasts for the life of the host, and although all infected individuals will develop histologic gastritis only a subset will develop symptomatic gastritis, peptic ulcer disease, gastric MALT lymphoma, or gastric adenocarcinoma. The bacterial and host factors that determine clinical outcome and influence the development of widely varying diseases have not been elucidated. We compared disease in *Helicobacter*-infected severe combined immunodeficient (SCID) mice on different genetic backgrounds with their corresponding immunocompetent partners to determine if the genetics of the host significantly impacts the innate inflammatory outcome, independent of variations in bacterial virulence factors. BALB/c SCID and C57BL/6 SCID mice developed equivalent histologic gastritis by 8 weeks of infection. Immunocompetent BALB/c mice and C57BL/6 mice developed significantly lower or higher degrees of inflammation respectively. Innate inflammation in immunodeficient mice on the C57BL/6 background remained low even in the absence of the regulatory cytokine IL-10. These results demonstrate that adaptive immunity is not required for the generation of low level inflammation in response to *Helicobacter* infection and that the degree of inflammation is consistent among different genetic backgrounds. Additionally, this inflammation is limited even in the absence of regulatory T cells.

## Introduction

Infection of the gastric mucosa by *Helicobacter pylori* (*H. pylori*) can result in a variety of distinct pathologic outcomes including symptomatic gastritis, peptic ulcer disease, gastric MALT lymphoma, and gastric adenocarcinoma. The majority of infected individuals remain healthy and asymptomatic but do develop histologic gastritis (Dooley et al., 1989). The mechanism(s) by which *H. pylori* induces distinct clinical outcomes and varying degrees of gastritis between individuals remain ill-defined. The diversity of *H. pylori*-associated diseases and severity can not be explained solely on the basis of specific virulence factors, although certain allelic variants of such genes as *cagA*, *vacA*, *oipA*, and *dupA* are attributed to more severe disease (Atherton et al., 1995; Blaser et al., 1995; van Doorn et al., 1998; Yamaoka et al., 2002; Amieva and El-Omar, 2008; Yamaoka, 2010). Similarly, factors such as diet or geography seem to be correlative of disease frequency in certain populations but these associations are not universal.

The host itself can play an important role in *H. pylori*-associated pathogenesis. This is illustrated by the association of specific IL-1β gene cluster polymorphisms that are linked not only to gastric cancer, but to *H. pylori*-associated hypochlorhydria (El-Omar et al., 2000; Machado et al., 2001). Additional studies have described a high risk for developing gastric cancer in patients infected with cagA-positive *H. pylori* that also expressed certain vacA alleles and who also possessed specific IL-1β and IL-1R polymorphisms (El-Omar et al., 2003). Studies on gastric biopsies from *H. pylori*-infected patients confirm that these IL-1β and IL-1R polymorphisms are associated with increased IL-1β production and gastric inflammation (Hwang et al., 2002). Polymorphisms in other host genes such as TNFα, IL-10, IL-8, and some TLRs have also been reported to influence *H. pylori* pathogenesis (Amieva and El-Omar, 2008).

Several studies in mice provide evidence for a significant role of host genetics in determining the severity of inflammation and histologic pathology during a gastric *Helicobacter* infection (Dooley et al., 1989; Mohammadi et al., 1996; Sakagami et al., 1996; Sutton et al., 1999; van Doorn et al., 1999; Sutton et al., 2000; Panthel et al., 2003). Whereas C57BL/6, SJL, and several other strains of mice have been shown to respond to a *H. felis* infection with moderate to high-grade inflammation, BALB/c, and CBA mice lacked inflammation and have been termed non-responders (Mohammadi et al., 1996; Sakagami et al., 1996). The variation in the degree of gastritis, at least between C57BL/6 and BALB/c mice could not be attributed to differences in MHC antigen expression as demonstrated through the use of congenic strains of mice (Mohammadi et al., 1996). Sakagami et al. (1996) demonstrated that mice such as C3H/He and CBA which share the same MHC haplotype develop differing levels of gastritis as do BALB/C and DBA/2 strains, also of identical haplotype. Sutton et al. (1999) later demonstrated that the non-responsive phenotype was dominant by examining the F1 progeny of the non-responder CBA mice crossed with either C3H/He, C57BL/6, or SJL mice compared to the parent strains when infected with *H. felis*. These studies were extended to *H. pylori* infection where it was demonstrated that non-responsiveness was associated with IL-10 production, indicative of an active immunosuppression by the host (Sutton et al., 2000).

Severe combined immunodeficient (SCID) or rag knockout mice generally lack *Helicobacter*-associated inflammation relative to immunocompetent mice (Eaton et al., 1999; Roth et al., 1999). However, we have demonstrated that C.B-17 SCID mice do develop a low grade response when infected with *H. felis* (Blanchard et al., 1995). The C.B-17 mouse has a BALB/c genetic background. Since SCID mice can be obtained on the high responder C57BL/6 background as well, we were interested in determining whether infection of these mice would result in increased *Helicobacter*-associated gastritis in the absence of adaptive proinflammatory or immunoregulatory T cells responses. The goal of the present study therefore, was to determine if the genetics of the host, independent of adaptive immunity, influence the degree of *Helicobacter*-induced inflammation, and to investigate the importance of IL-10 in limiting gastric inflammation.

## Materials and Methods

### Mice

Six- to eight-week-old C57BL/6 Rag1 deficient mice, C57BL/6 IL-10 deficient mice, SCID mice on either C57BL/6 or BALB/c backgrounds, as well as congenic immunocompetent C57BL/6 and BALB/c wild type mice were purchased from Jackson Laboratory (Bar Harbor, ME). Mice double deficient in Rag1 and IL-10 were generated by crossing the respective transgenic strains and subsequent progeny. Mice were screened for the appropriate mutations by PCR on genomic DNA using oligonucleotide primers and protocols as specified by Jackson Laboratories. Mice homozygous for both mutations were used for the studies described below. All mice were housed in microisolater cages and fed autoclaved laboratory chow and sterile water *ad libitum* to minimize the introduction of exogenous bacteria at either Case Western Reserve University or the University of Maryland at Baltimore animal facilities. Both facilities are fully accredited by the American Association for Accreditation of Animal Care International, and all studies involving the use of mice were reviewed and approved by the relevant Institutional Animal Care and Use Committees of Case Western Reserve University or the University of Maryland Baltimore.

### Bacteria

*Helicobacter felis* was isolated from a feline gastric biopsy specimen at the Case Western Reserve University as previously reported, (Czinn et al., 1993) and identified as *H. felis* based on colony and bacterial morphology, Gram stain, and the production of urease, catalase, and oxidase (Lee et al., 1990). *H. felis* was maintained on Columbia blood agar (Difco, Detroit, MI) containing 7% horse blood under microaerobic conditions (5% O_2_, 10% CO_2_) at 37°C for 96 h and used for the production of whole cell lysate antigen and to infect mice.

### *Helicobacter* lysate

Lysate was prepared as previously described (Czinn et al., 1993). Briefly, *H. felis* bacteria were harvested in sterile PBS and washed by centrifugation at 4000 *g* for 20 min. Cells were re-suspended in PBS and lysed using a probe sonicator (Sonics and Materials Inc., Danbury, CT, USA) set at 50% duty cycle and a power setting of 5. Remaining whole cells were removed by centrifugation at 400 *g* for 20 min and filtering the supernatant through a 0.22 μM pore filter. The protein concentration was determined by the Lowry assay (Lowry et al., 1951).

### Treatment of mice

Infection of mice was accomplished by oral inoculation with approximately 5 × 10^7^ *H. felis* on two consecutive days. Oral inoculation was accomplished by gastric intubation using flexible tubing placed on the end of 18 g needles.

### Histologic analysis of the gastric mucosa

A continuous strip of tissue was surgically removed from the greater curvature of the stomach of each mouse and fixed in 10% buffered formalin. H&E stained sections were prepared from paraffin-embedded tissue. Inflammation of the antral and fundic tissue were determined in blinded fashion as previously described (Blanchard et al., 1995). Samples were assigned a global score from 0–10 (fundus) or 0–3 (antrum) based on a combination of the intensity (in the most severe 10× microscopic field), extent (percentage of the mucosal surface involved), and depth (basal, panmucosal, submucosal) of inflammation.

### Bacterial load determination

*H. felis* often grows as confluent colonies making it difficult to determine CFUs. Therefore, for initial experiments comparing SCID and wild type mice, tissue sections prepared from the paraffin-embedded biopsies described above were silver stained using the Steiner’s stain method to facilitate identification of *H. felis* by microscopic observation. Bacterial load was calculated by determining the number of *H. felis* positive glands divided by the length of the sample using an eyepiece with a calibrated grid. The bacterial load for the subsequent experiment on IL-10 and rag1 deficient mice was determined by quantitative PCR. Briefly, total DNA was extracted from frozen gastric biopsy strips using DNeasy (Qiagen) using the recommended modification to optimize release of bacterial DNA from tissue bound bacteria. PCR amplification was performed on an Eppendorf Realplex real time thermocycler (Westbury, NY) using primers specific for 16S rRNA (Kong et al., 1996). For each sample the PCR reaction was performed in triplicate with the SYBR Green supermix (Fermentas, Glen Burnie, MD, USA) and compared to a standard curve of known amounts of chromosomal *H. felis* DNA.

### Anti-*Helicobacter* ELISA

Serum anti-*Helicobacter* IgG titers were determined by ELISA as previously described (Blanchard et al., 1995). Briefly, 96 microtiter plates (Nalge Nunc International, Rochester, NY, USA) were coated with 100 μl *H. felis* lysate antigen at 10 μg/ml overnight, blocked for 1 h with 200 μl 1% bovine serum albumin in PBS and then incubated for 1 h in sequence with 100 μl/well of serum dilutions, goat anti-mouse IgG conjugated to alkaline phosphatase (Jackson Immunoresearch Laboratories Inc., West Grove, PA, USA) and then developed with 100 μl/well *p*-nitrophenyl phosphate substrate (Sigma Aldrich, St. Louis, MO, USA). Wells were washed between steps three times with PBS containing 0.05% Tween 20. Optical density was determined at 405 nm. Titers were determined as the greatest dilution of serum giving a reading of at least 0.05 OD greater than our conjugate control.

### Cell proliferation assay

Spleens were surgically removed from mice at sacrifice and used to make single cell suspensions. Red blood cells were lysed and cells were distributed at 1 × 10^6^ cell/well in 96 well flat bottom plates in HL-1 media containing 2 mM _l_-glutamine. Cells were incubated with either 10 μg/ml *H. felis* antigen (previously optimized) or 1 μg/ml Conconavalin A mitogen for 5 days. Cells were pulsed with 1 μCi ^3^H-thymidine per well for the final 16 h. ^3^H-thymidine incorporation was determined by liquid scintillation counting.

### Statistical analysis

Data representing antibody titers, bacterial load, and inflammation are expressed as means ± standard deviation and comparisons between experimental groups was evaluated by ANOVA.

## Results

### *Helicobacter* induces more gastric inflammation in SCID mice than wild type BALB/c mice

**Figure 1:**
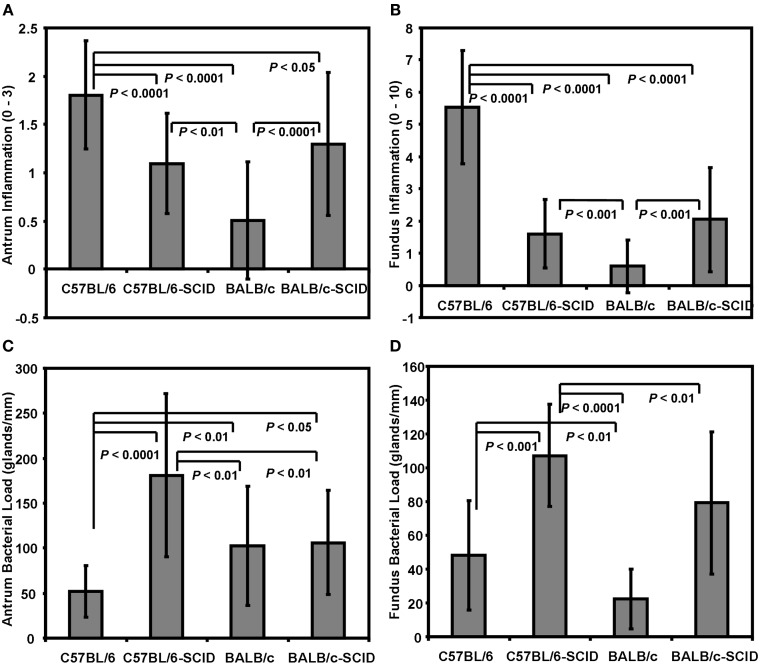
**Immunodeficient SCID mice develop more *Helicobacter*-associated gastric inflammation than Balb/c counterparts**. C57BL/6, and BALB/c mice along with their SCID counterparts were (infected) with *H. felis* and then evaluated for inflammation in both the **(A)** antrum and **(B)** fundus by histologic grading of H&E stained longitudinal sections. The bacterial load for both the **(C)** antrum and **(D)** fundus were quantified by direct visualization of Steiner (silver) stained section and determining the number of *H. felis* infected glands per mm of tissue. *N* = 8–12 mice per group.

In two separate experiments, 8–12 BALB/c and C57BL/6 mice along with their SCID counterparts were orally inoculated with *H. felis* and sacrificed 8 weeks post infection. Upon sacrifice all mice inoculated with *H. felis* were determined to be positive for infection by either the urea test broth assay and or by culture of organisms from gastric biopsies. As expected, C57BL/6 mice developed histologic gastritis that was significantly more severe than that of BALB/c mice in both the antrum (*P* < 0.0001) and fundus (*P* < 0.0001; Figures 1A,B). These findings are consistent with the previously reported high and low responder status of these animals (Mohammadi et al., 1996; Sakagami et al., 1996). SCID mice of each background responded to infection with mild inflammation in both the antrum and fundus and there was no difference between the two SCID strains. The degree of gastric inflammation compared to wild type C57BL/6 mice was significantly lower for both strains of SCID mice in both the antrum and fundus. The low responding BALB/c mice however demonstrated significantly less inflammation than the C57BL/6 SCID mice in both the antrum (*P* < 0.0001) and fundus (*P* < 0.0001), as well as compared to BALB/c SCID mice in the both the antrum (*P* < 0.001) and fundus (*P* < 0.001).

### High responder C57BL/6 mice have a reduced bacterial load

There was a general trend for reduced bacterial counts in the gastric mucosa of mice with more severe inflammation (Figures 1C,D). When the number of infected glands per mm of tissue was evaluated in the antrum, the high inflammation wild type C57BL/6 mice had significantly fewer infected glands then both the C57BL/6 SCID (*P* < 0.0001), BALB/c SCID (*P* < 0.05), as well as the wild type BALB/c mice (*P* < 0.01; Figure 1C). The number of antral infected glands in the BALB/c mice was similar to that of the BALB/c SCID mice with average counts of 103 ± 66 and 106 ± 58 respectively, but lower than the C57BL/6 SCID mice (*P* < 0.01). Bacterial load for the fundus however revealed significantly lower infected glands in both C57BL/6 and BALB/c mice compared to their respective SCID counterparts (Figure 1D; *P* < 0.001 and *P* < 0.01 respectively) and the C57BL/6 and BALB/c SCID groups harboring statistically similar loads (107 ± 30 and 79 ± 42 respectively).

Severe combined immunodeficient (SCID) mice have the potential to become “leaky” as they age, developing some functional B and T cells. To rule out the possibility that the low level inflammation observed in the SCID mice was due to such leakiness, *H. felis*-specific IgG titers were determined from sera collected at sacrifice (data not shown). All BALB/c mice lacked specific IgG titers while C57BL/6 mice developed titers averaging 1:14,265 ± 10,776. C57BL/6 SCID mice also failed to generate any *H. felis*-specific IgG. Low titers of less than 1:1000 were observed in a subgroup of seven BALB/c SCID mice but only in one of the two experiments and these mice developed equivalent levels of inflammation as the mice with no detectable antibodies. However, in spleen cell proliferation assays, whereas wild type C57BL/6 and BALB/c mice responded to antigen stimulation compared to cells exposed to media alone (*P* = 4 × 10^−7^ and *P* = 0.043 respectively), there was a lack of ^3^H-thymidine uptake by SCID mice of either background (data not shown).

### Lack of IL-10 does not increase *Helicobacter*-associated gastritis in the absence of adaptive immunity

**Figure 2:**
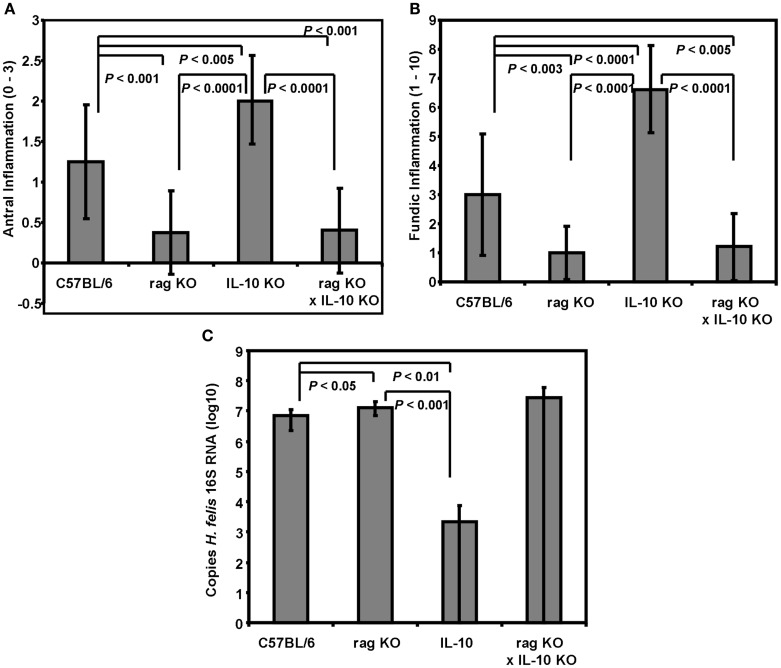
**Immunodeficient rag1 ^−/−^ mice do not develop significant *Helicobacter*-associated gastric inflammation even in the absence of IL-10**. C57BL/6 as well as rag1^−/−^, IL-10^−/−^, and rag1-IL-10 double knockout mice all on C57BL/6 backgrounds were infected with *H. felis* and then evaluated for inflammation in both the **(A)** antrum and **(B)** fundus by histologic grading of H&E stained longitudinal sections. The bacterial load per stomach was determined by quantitative PCR of the 16S RNA gene from a single strip biopsy encompassing the entire length of the gastric mucosa **(C)**. Data is reported as DNA copies per gram stomach tissue. *N* = 8–10 mice per group.

Previous reports indicate that SCID and rag deficient mice on the C57BL/6 background are lacking gastric inflammation following infection with *H. felis* and *H. pylori* (Eaton et al., 1999; Roth et al., 1999). The experiments described above revealed an intermediate amount of inflammation for SCID mice that was greater than that of BALB/c mice indicating that adaptive immunity is not required to generate a low level of *Helicobacter*-induced gastritis. The reduced amount of gastritis in SCID mice compared to C57BL/6 mice however, suggests there may be down-regulatory elements that prevent the mouse from mounting a more significant innate response. One such down-regulatory element could be IL-10. Although lymphocytes are thought to be the primary source of IL-10, other cell types including monocytes/macrophages, dendritic cells, mast cells, and intestinal epithelial cells have also been reported to produce IL-10 under a variety of *in vivo* and *in vitro* conditions (Moore et al., 2001; Ma et al., 2003; Drakes et al., 2004; Drakes et al., 2006; Gee et al., 2006; Grimbaldeston et al., 2007). Therefore, since IL-10 is known to play an important role in limiting the adaptive immune and inflammatory response to both *H. felis* and *H. pylori*, (Berg et al., 1998; Chen et al., 2001; Eaton et al., 2001; Ismail et al., 2003; Matsumoto et al., 2005) we developed *rag*1 x IL-10 double knockout mice to determine if *H. felis* infection would induce a more significant innate inflammatory response in these mice. Control wild type C57BL/6 mice responded to *H. felis* infection with average gastritis scores of 1.25 ± 0.7 and 3.0 ± 2.0 in the antrum and fundus respectively (Figures 2A,B). IL-10 deficient mice developed significantly greater inflammation than C57BL/6 mice in the antrum (*P* = 0.003 and fundus (*P* < 0.001) as expected based on previous reports (Ismail et al., 2003; Matsumoto et al., 2005). The *rag*1^−/−^ mice developed mild inflammation that was significantly less than the C57BL/6 mice in both the antrum (*P* = 0.0008) and fundus (*P* = 0.026). Mice deficient in both *rag*1 and IL-10 were equivalent to *rag*1^−/−^ mice. There was a wide variation in bacterial loads within the IL-10^−/−^ and the rag1^−/−^ × IL-10^−/−^ mice (Figure 2C). No significant differences in loads were observed between C57BL/6, *rag*1^−/−^, and *rag*1^−/−^ × IL-10^−/−^ mice except between *rag*1^−/−^ and C57BL/6 mice (*P* = 0.033). IL-10^−/−^ mice had significantly fewer bacteria than both *rag*1^−/−^ mice (*P* < 0.001) and C57BL/6 mice (*P* < 0.01).

## Discussion

These results indicate that host genetics are of limited influence on the innate inflammatory response to gastric *Helicobacter* infections in the absence of adaptive immunity. T cells are necessary to promote the panmucosal, chronic-active gastritis typically associated with gastric *Helicobacter* infections, (Eaton et al., 1999; Roth et al., 1999) but we previously demonstrated that immunodeficient SCID mice develop low grade inflammation in response to *H. felis* infection (Blanchard et al., 1995). The current data demonstrate that in the absence of adaptive immunity, the inflammatory response between C57BL/6 and BALB/c SCID mice is equivalent. Wild type BALB/c mice however responded to infection with a significantly weaker response than their corresponding SCID animals while wild type C57BL/6 mice generated significantly greater inflammation than the corresponding SCID mice. The failure of the immunodeficient mice to generate more severe inflammation is not due to the presence of the down-regulatory cytokine IL-10 as *rag*1 × IL-10 double knockout mice responded similar to *rag*1 deficient mice.

Inbred strains of mice have been used previously to demonstrate that the genetics of the host determines whether a strain will be a high or low responder to *Helicobacter* infection as determined by the degree of gastric inflammation (Mohammadi et al., 1996; Sakagami et al., 1996; Sutton et al., 1999; van Doorn et al., 1999; Sutton et al., 2000; Panthel et al., 2003). C57BL/6, SJL, and A/J mice have been demonstrated to be high responders whereas BALB/c and CBA mice have been termed low or non-responders (Mohammadi et al., 1996; Sakagami et al., 1996 and unpublished data). Non-responsiveness is dominant in the F1 progeny of high and non-responder crosses, and IL-10 seems to play an important role in limiting the inflammation in the non-responding animals (Sutton et al., 1999, 2000). The importance of IL-10 in limiting the host response has been confirmed in other models in which IL-10 deficient mice chronically infected with either *H. felis* or *H. pylori* develop pronounced histologic inflammation that reduces or eradicates the bacteria from the gastric mucosa (Berg et al., 1998; Chen et al., 2001; Eaton et al., 2001; Ismail et al., 2003; Matsumoto et al., 2005).

There are now many reports documenting the importance of regulatory T cells in limiting the host response to gastric *Helicobacter* infection (Lundgren et al., 2003; Raghavan et al., 2003; Raghavan et al., 2004; Lundgren et al., 2005; Anderson et al., 2006; Rad et al., 2006; Harris et al., 2008; Kao et al., 2010; Arnold et al., 2011; Sayi et al., 2011). Depletion of Treg cells, or limitation of their activity, results in significantly more severe gastritis that typically results in reduced bacterial loads in the gastric mucosa. These cells have been identified in human gastric biopsies as well, and they have been shown to suppress the *H. pylori*-specific recall response *in vitro* (Lundgren et al., 2003; Harris et al., 2008). The present study indicates that even in the absence of such down-regulatory T cells, the host has a measurable but limited inflammatory response, regardless of host genetics.

Several factors may contribute to the failure of mice lacking mature T and B lymphocytes to mount a severe granulocyte and monocyte based inflammatory response in the absence of down-regulatory T cells. First, *H. pylori* and *H. felis* lack pathogen associated molecular pattern (PAMP) molecules with high activity and therefore may simply not be very effective at activating innate cells *in vivo*. The LPS molecules of *H. pylori* and *H. felis* have structural features such as tetra-acetylated Lipid A that severely limit their ability to activate cells through the TLR4 receptor (Moran, 1996; Moran et al., 1997). Similarly, the amino acid sequence on the flagella domain known to interact with TLR5 is distinct from that of other bacterial flagella molecules resulting in a failure to activate cells through TLR5 (Gewirtz et al., 2004; Andersen-Nissen et al., 2005). Although TLR2 has been shown to be a major PAMP for *Helicobacter* antigens, (Rad et al., 2009; Sayi et al., 2011). TLR2 has also been shown to play a role in limiting the *Helicobacter*-induced inflammatory response (Sayi et al., 2011). *H. pylori* peptidoglycans can interact with NOD1 to promote NFκB activation and proinflammatory events (Viala et al., 2004). Such activity however requires transport to the host cell cytoplasm via the *H. pylori* Type IV secretion system which is non-functional in *H. pylori* SS1 (Crabtree et al., 2002; Philpott et al., 2002) and absent in *H. felis*.

It is also likely the host itself limits the inflammatory response to environmental challenge at mucosal surfaces. Thus, even in the absence of regulatory T cells, if T helper cell activity is also absent the host might be predisposed to limit inflammation in these tissues. This is consistent with the lack of colitis typically observed in SCID or rag deficient mice which are colonized with commensal organisms. Even when *rag*1^−/−^ mice are colonized with *Citrobacter rodentium* which typically causes colonic hyperplasia in mice, the acute phase granulocyte based colitis was transient and by 21 days returned to normal, despite increasing numbers of colonizing bacteria (Vallance et al., 2002). In our model, mice lacking both T cells and IL-10 remained healthy. Therefore some other as yet undescribed mechanisms must play an active role in limiting acute inflammation in the gastrointestinal tract. TGFβ may contribute since it is also produced by a variety of cells and is immunosuppressive. Thus an IL-10 mediated T cell regulation as well as a separate innate regulation mediated through TGFβ or some other factors contribute to the immunologic quiescence in the mucosa. It is interesting to note that when regulatory T cells are present, they effectively suppress not only the helper T cell induced inflammation but the innate inflammatory response as was evident when we compared wild type BALB/c mice with the corresponding SCID mice on the BALB/c background.

These data provide additional evidence that IL-10 secreting iTreg cells or Tr1 regulatory T cells play an active role in limiting the host response to gastric infection with *Helicobacter* bacteria but also demonstrate that mice deficient in adaptive immunity do develop innate gastritis that is independent of host genetics. Additional studies in this and other models will be necessary to elucidate the host mechanisms employed to limit this response in the absence of regulatory T cells.

## Conflict of Interest Statement

The authors declare that the research was conducted in the absence of any commercial or financial relationships that could be construed as a potential conflict of interest.
